# Impact of ageing on discourse types: a comparative study

**DOI:** 10.1590/2317-1782/e20250028en

**Published:** 2026-03-27

**Authors:** Deepak Puttanna, Darshan Hosaholalu Sarvajna, Melemane Kalappa Niharika, Anisha Michel Sequeira, Jenin Dsouza

**Affiliations:** 1 Department of Audiology and Speech-Language Pathology, College of Medical Rehabilitation Sciences, Taibah University - Al-Medinah, Saudi Arabia.; 2 Nitte Institute of Speech and Hearing - NISH, Nitte (Deemed to be University) - Mangalore, India.; 3 JSS Institute of Speech and Hearing - Mysuru, India.; 4 Pragyan Child Development Centre - Basavanagudi (Bangalore), India.; 5 Department of Speech and Hearing, Father Muller College - Mangalore, India.

**Keywords:** Narration, Ageing, Language Production, Conversation, Discourse, Bilingualism, Discourse Analysis Scale

## Abstract

**Purpose:**

Ageing is associated with a decline in physical, physiological, and cognitive-linguistic abilities, impacting language and social participation. Discourse, a critical aspect of language, involves the integration of phonology, lexicon, syntax, cohesion, and coherence. Examining variations in discourse types is essential for understanding age-related changes and improving language assessment and treatment strategies. Therefore, the present study investigated the effect of ageing on discourse production across different discourse types.

**Methods:**

This study recruited 120 neurotypical, Konkani-Kannada bilingual participants. Participants were divided into three age groups: young adults (18–35 years), middle-aged adults (35.1–55 years), and older adults (55.1–70 years). Discourse tasks included picture description, narration, and conversation, conducted in Konkani, their native language. Responses were digitally recorded, transcribed, and analyzed using the Discourse Analysis Scale. Discourse Quotient was calculated for each task through qualitative analysis.

**Results:**

Significant differences in discourse performance were observed across all three types among the three groups. Additionally, significant differences were noted across discourse types within young, middle, and older adults. However, older adults exhibited a different pattern of performance, performing poorer on picture description compared to the other two types and their counterpart groups.

**Conclusion:**

The study highlights the influence of ageing and task type on discourse production, with older adults facing greater challenges in narrative tasks. These findings emphasize the need for speech-language pathologists to consider the linguistic and cognitive demands of older adults in clinical interventions, enabling the development of more effective language assessment and therapy programs.

## INTRODUCTION

Understanding the intricacies of human ageing is a complex task. This complexity is further intensified when we attempt to determine how these natural processes impact human social communication. A decline in physical, physiological, and cognitive performance generally accompanies ageing. These changes influence social participation and quality of life, especially where older adults are concerned; therefore, examining language changes is of utmost importance^(1)^. Discourse is one such aspect of language that defines the social construction of ideas, based on values, beliefs, and culture. Discourse comprises a set of utterances to convey a message and is the most elaborate linguistic activity^(2)^. Discourse production simultaneously activates the various linguistic components such as phonology, lexicon, syntax, cohesion, and coherence^(3)^. It requires an appropriate combination of different processing stages: the within-sentence micro-linguistic aspects and the between-sentence macro-linguistic processes^(4)^.

Given the effects of ageing on micro-linguistic and macro-linguistic aspects of discourse, older adults often experience difficulty in lexical selection, morphological, and syntax processing^(5,6)^. The macro-linguistic aspects of discourse, including cohesion, coherence, and completeness, are found to decline with age^(7)^. However, a few micro-linguistic aspects, such as lexical diversity, remain resistant to declining age^(8)^. Fergadiotis and Wright^(8)^ reported that discourse types influence lexical diversity. For example, older adults showed constrained discourse in elicitation tasks using pictorial stimuli (such as picture description) compared to procedural and recount discourse that utilized non-pictorial stimuli. Similarly, Wright and Capilouto^(9)^ reported a consistent decline in coherence and other macro-linguistic aspects across discourse types. A majority of studies have examined narrative discourse^(8-10)^ and compared it with other types such as recounts, picture descriptions, procedural, and conversational discourse^(11,12)^. The discourse abilities of older adults differed from those of young adults in terms of communication engagement and initiative, number of words per minute, correct information units, and overall communicative goals^(12)^. However, these findings are inconsistent, given the effects of age on discourse production across different types.

Discourse production is subserved by other cognitive-linguistic mechanisms, including attention, semantic memory, episodic memory, working memory, and executive functions that change as individuals age^(13)^. Additionally, individual factors such as education have demonstrated positive effects on oral discourse production during typical ageing^(14,15)^. The rules and features that govern discourse vary across languages, leading to differences in performance across the languages assessed. For example, discourse production has been studied in English^(11),^ Persian^(16),^ and Italian^(6)^. Given the linguistic and socio-cultural differences, it's essential to study discourse in various languages. As a multilingual and multicultural country, India provides a unique setting to explore language behaviors, especially in the context of discourse. Investigating discourse production among the older population is crucial, considering their expected growth and increased vulnerability to communication disorders among older adults^(17,18)^. In this line, John et al.^(18)^ investigated narrative and procedural discourse as a function of ageing among Malayalam speakers. They found older adults to have used more words, rephrased the sentences, and had longer mean length of utterances, resulting in a more elaborate speech as compared to young adults. Conversely, the discourse of young adults was more structured and organized with complex sentences. On comparing various discourse types, the performance in narrative discourse was higher than in procedural discourse. Nonetheless, other languages of India remain unexplored, which otherwise would have major clinical implications in the assessment and management of age-related communication disorders^(19)^. Given the significant growth of bilingual populations worldwide and in India, it's essential to establish norms based on bilingual individuals. Bilingualism is known to change the brain's structure and function by constantly recruiting and inhibiting unintended languages during language production^(20)^. This led the authors to explore discourse production in bilingual individuals, including Konkani-Kannada bilinguals. There is currently no data on discourse production in bilingual populations, particularly those speaking Konkani and Kannada. In Karnataka state, approximately one million people speak Konkani and Kannada. To effectively serve the clinical population in this cultural context, there is a need to establish culturally sensitive norms from this community. Hence, the present study aimed to determine the effect of age on different types of discourse. To achieve this aim, the authors designed two main research questions;

1. Does discourse performance (conversation, narration, and picture description) vary across young adults, middle-aged adults, and older adults?
2. Does discourse performance vary across types (conversation, narration, and picture description) within each age group (young adults, middle-aged adults, and older adults)?

## METHODS

### Participants

In the present study, a total of 120 neurotypical Konkani-Kannada bilingual individuals were recruited using a convenience sampling method. All participants were residents of the Mangalore district in the state of Karnataka, India. Recruitment was facilitated through local community networks and organizations to ensure broad representation within the bilingual population.

Strict inclusion criteria were applied to ensure participant suitability. Only individuals with high proficiency in both Konkani and Kannada were included. All participants were simultaneous bilinguals who rated their proficiency in both languages as equally strong, based on the modified language proficiency scale developed by Amruthavarshini and Yathiraj^(21)^. Additionally, only those with a minimum educational qualification of matriculation were considered eligible for participation in the study.

Further, they were divided into three groups, namely young adults (18-35 years), middle-aged adults (35.1- 55 years), and older adults (55.1 to 70 years). Demographic details of the participants are given in Table 1. The Mann-Whitney U test results revealed no statistical difference in age between males and females in the young adults’ group (*U*=53.00, *p*=0.36), middle-aged adults (*U*=101.00, *p*=0.36), and older adults (*U*=173.5, *p*=0.48).

**Table 1:** Gender and educational details across young, middle, and older adult groups

	**Group**	**Young**	**Middle**	**Old**
**Gender Distribution**	Male	05%	20%	47.5%
	Female	95%	80%	52.5%
**Age (in years)**	Male	19.5 (0.70)	37.5 (3.58)	58.8 (8.70)
	Female	21.1 (2.81)	36.6 (4.71)	57 (8.76)
**Education**	Pursuing Under-graduation	67.5%	-	-
	Graduation	30%	95%	70%
	Pre-University education	2.5%	2.5%	15%
	Matriculation	-	2.5%	15%

The study followed the “Ethical Guidelines of Bio-Behavioral Research Involving Human Subjects” and was approved by the Institutional Ethical Board with protocol number FMIEC/249/2024. Informed consent was taken from each participant before the administration of the tasks.

### Measures and procedure

The Montreal Cognitive Assessment^(22)^ was used to exclude the participants who had a cognitive impairment, and the participants who scored < 26 were not recruited in the study. Also, the WHO Ten Question Screening^(23)^ was administered to all participants to ascertain their history of sensory, developmental, learning, or neurological deficits, and to exclude them if any such history was mentioned.

The assessment comprised three distinct tasks, namely, picture description, narration, and conversational exchange. All these tasks were administered in the participants' native language, Konkani (L1), to ensure natural and accurate language use.

The picture description task was carried out using a standardized picture stimulus. Here, participants were presented with a series of three standardized picture sequences: *Broken Window*, *Cat Rescue*, and *Refused Umbrella*^(24)^. Each picture sequence was shown individually, and participants were allotted one minute to observe the images before initiating their description. The instruction provided was: *"Take a look at the pictures, you will be given one minute to view each picture, and then you will be asked to describe the story depicted in the sequence. Try to construct a narrative with a clear beginning, middle, and end."* This task aimed to assess the participant's ability to organize visual stimuli into a coherent and structured verbal narrative.

In order to gather narrative discourse samples, participants were asked to recall and describe a personal past incident. The specific instruction given in this task was, "Please narrate a personal story or experience from your past that is meaningful to you." It was ensured that a sufficiently detailed discourse sample was generated by asking participants to speak for at least two minutes. Without changing the narrative's substance or structure, the examiner played a non-directive role, using only brief cues like "Anything else?" or vocal affirmations (such "Okay," "Hmm") to urge the story to continue.

The participant and examiner engaged in a semi-structured conversation to elicit conversational discourse. Naturalistic speech production was facilitated, and pragmatic language use was evaluated using a series of open-ended, daily enquiries. Some examples were: "What is your usual morning routine?”, "What do you typically do during your free time?”, plus "Can you tell me about your hobbies or interests?" To keep participants interested and encourage natural discourse, the examiner modified follow-up questions in response to their answers.

### Data analyses

The digital recording of the discourse samples was followed by transcription for in-depth examination. Both propositional and non-propositional features of communication were assessed using the Discourse Analysis Scale (DAS)^(25)^, which was used to evaluate these transcriptions. Repair techniques, revision behaviours, and turn-taking patterns were examples of non-propositional elements, whereas propositional elements comprised characteristics like discourse structure, communication intent, and subject management. On a standardized three-point scale, with 0 indicating poor, 1 indicating fair, and 2 indicating good, each discourse sample was evaluated for all elicitation tasks. This approach allowed for the systematic evaluation of discourse quality in both content and interactional dimensions. The robustness of the DAS scale involves evaluating discourse in terms of propositional and non-propositional aspects of communication, and the fact that the discourse sample can be evaluated using a variety of tasks, including picture description, narrative tasks, and conversation tasks, led to its adoption in the current study^(26)^.

## RESULTS

The data from 120 participants, distributed across three groups: young adults, middle-aged, and older adults, was analyzed. The study groups exhibited no statistically significant differences in terms of age, gender, or education. Initially, the data underwent a Shapiro-Wilk test to assess its normality. The test results indicated a significant deviance from normality (*p*<0.05).

Descriptive statistics were computed, considering the median and interquartile range (IQR). Kruskal-Wallis and Mann-Whitney tests were employed for further analysis. The data analysis was performed using JASP software (version 0.17.2.1), and statistical significance was set at an alpha of less than 0.05. In addition, the authors computed effect size (re)= Z/√N for the computed Z scores, where N denotes the total number of observations^(27)^.

### Between-group analysis across various discourse types

**Figure 1:**
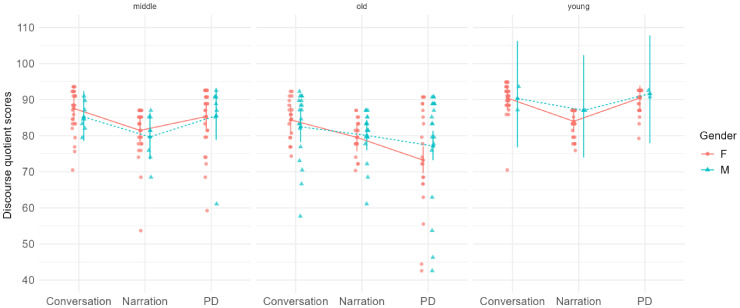
Discourse quotient scores across discourse types and across the age groups

Upon descriptive statistics, the Median and IQR were computed. The median scores and IQR for all three discourse types across the three groups are tabulated (See Table 2). The graphical representation of Discourse Quotient (DQ) scores across age groups, gender, and tasks is depicted in Figure 1.

**Table 2:** Median and Interquartile range values of Discourse Quotient across conversation, narration, and picture description types in young, middle, and older adults.

	**Conversation**			**Narration**			**Picture description**		
	**Young**	**Middle**	**Old**	**Young**	**Middle**	**Old**	**Young**	**Middle**	**Old**
Median	91.02	88.46	85.89	87.03	83.33	81.48	92.59	88.88	79.62
IQR	5.12	8.97	10.26	4.16	7.87	5.56	3.71	7.87	20.83

**Caption:** PD- Picture Description, F- Female, M- Male

To investigate the research question of whether discourse types vary across the three groups, a Kruskal Wallis- H test was performed. The results of the Kruskal Wallis- H test showed discourse scores in conversation tasks were significantly different across three age groups, *H*(2)=25.27, *p*<0.05, and between genders, *H*(1)=8.51, *p=0.004*. Pairwise comparisons using Dunn’s post hoc showed DQ in conversation differed significantly between possible pairs of groups (young vs middle vs older) (*p<*0.05*)*. In addition, effect size was calculated for the possible pairs (See Table 3).

**Table 3:** Significance levels of discourse scores in conversation tasks in different age groups

**Groups**	***Z***	***Wi***	***Wj***	***P***	***Re***
Middle – Old	2.235	59.087	41.775	0.025^*^	0.24
Young - Middle	2.782	59.087	80.638	0.005^**^	0.30
Young–Old	5.018	41.775	80.638	< .001^**^	0.56

Effect size (re) if<0.3 was considered as low; 0.3-0.5 was considered as medium; and > 0.5 was considered high

* *p*<0.05,

** *p*<0.01,

Similarly, in the narration task, there was a significant difference across three age groups, *H*(2)=14.49, *p*<0.001, and no difference between genders, *H*(1)=1.33, *p = 0.24.* Dunn’s post hoc results showed DQ in narration differed significantly between young vs middle and young vs older adults (*p<*0.05*)*. The effect sizes for the possible pairs of groups are presented (See Table 4).

**Table 4:** Significance levels of discourse scores in the narration task in different age groups

**Comparison**	***Z***	***Wi***	***Wj***	***P***	***Re***
Middle – Old	1.528	58.65	46.98	0.12	0.17
Young -Middle	2.25	58.65	75.86	0.02^*^	0.25
Young–Old	3.78	46.98	75.86	< .001^**^	0.42

Effect size (re) if<0.3 was considered as low; 0.3-0.5 was considered as medium; and > 0.5 was considered high

* *p*<0.05,

** *p*<0.01

Between-group comparison on discourse scores in picture description found a significant difference, *H*(2)=41.97, *p*<0.01, and gender effect *H*(1)=3.97, *p=0.046.* Dunn’s post hoc showed DQ in picture description differed significantly between possible pairs of groups (*p<*0.05)*.* The effect size was large for younger vs older adults, but for the pair’s young vs middle-aged adult and middle vs older adult, a medium effect size was noted (See Table 5).

**Table 5:** Significance levels of discourse scores in picture description tasks in different age groups

**Comparison**	***Z***	***Wi***	***Wj***	***P***	***Re***
Middle – Old	3.37	61.17	35.32	<0.001^**^	0.37
Young - Middle	-3.10	61.17	85.00	0.002^**^	0.34
Young– Old	-6.47	35.32	85.00	< .001**	0.72

Effect size (re) if<0.3 was considered as low; 0.3-0.5 was considered as medium; and > 0.5 was considered high

** *p*<0.01

### Within-group analysis across different discourse types

On investigating the research question, whether the performance of discourse type varies among young adults, middle-aged, and older adults, the authors employed Friedman's test, followed by pairwise comparisons. Friedmann’s test showed that discourse types in the young adults’ group have a significant effect on DQ, *χ*^2^ (2) = 48.31, *p*<.001. Conover’s pairwise post hoc comparisons showed that DQ was higher in picture description (*Median*= 92.59) and was significantly higher than narration task (Median = 87.03: *T*(78) = 6.21, *p*<.001) and similar scores to conversation (Median = 91.02: *T*(78) = 0.39, *p*>.05). The DQ was higher in conversation compared to scores in narration *T*(78) = 5.81, *p*<.001).

Similarly, in the middle-aged group, a significant effect on DQ across discourse types was observed *χ*^2^ (2) = 22.39, *p*<.001. Conover’s pairwise post hoc comparisons showed that DQ was higher in picture description (Median= 88.9) and was significantly higher than narration (Median = 83.33: *T*(78) = 3.45, *p*<.001) and similar scores to conversation (Median = 88.46: *T*(78) = 1.07, *p*>.05). The DQ was higher in conversation compared to scores in narration *T*(78) = 4.53, *p*<.001).

In the older adults’ group, a significant effect on DQ was noted, *χ*^2^ (2) = 8.86, *p*<.005. Conover’s pairwise post hoc comparisons showed that DQ was higher in conversation (Median= 85.89) and was significantly higher than narration (Median = 81.48.33: *T*(78) = 2.81, *p*<.05) and picture description (Median = 79.62: *T*(78) = 2.25, *p<*.05). No significant difference between narration and picture description *T*(78) = 0.56, *p*>.05).

## DISCUSSION

The study aimed to unravel the impact of ageing on different types of discourse (conversation, narration, and picture description). The authors identified two critical findings. Firstly, older adults performed significantly poorer than middle-aged and younger adults in various discourse types, including conversation, narration, and picture description. This finding aligns with a plethora of studies^(6,16,28,29)^. It corresponds with the reports of Pereira et al^(12)^, which suggest that older adults provide more concrete and less reliable information than younger adults for a given topic. The speech of older adults was often disorganized, with incoherence and tangential utterances present compared to their younger counterparts^(30)^. In most instances, older adults were able to maintain the topic of conversation only with the help of cues provided by the examiner, which was also observed in our study. These findings support earlier research that elucidated functional impairments in speech associated with deficits in verbal inhibitory control^(30,31)^. In addition, the lack of cohesion and semantic relatedness noted in older adults was supported by findings of Saling et al.^(32)^

From the reports of Marini et al.^(6)^ conclusions about poorer performance in discourse types can be drawn. Specifically, behaviors related to lexical-semantic access, such as paraphasias and the repetition or reformulation of words and sentences, indicate that older adults exhibit difficulties in lexical-semantic access, which is more prevalent with ageing. Lexical, phonological, and morphological access are the features of micro-linguistic processing, all of which deteriorate with ageing. Moreover, a decline in cognitive functioning, such as working and long-term memory, is shown to be part of the ageing process^(33,34)^. Executive functioning skills may also be considered pivotal for discourse generation^(33,35)^. These findings provide insights into the poor organization of thoughts and the difficulty in formulating them into externalized verbal utterances, which can be attributed to reduced cognitive capacities, such as working memory, inhibitory control, and organization.

Furthermore, the use of the DAS scale in the current study facilitates the analysis of the participants' discourse abilities in terms of both propositional and non-propositional aspects. Propositional discourse elements encompass linguistic features such as temporal and causal relations (e.g., *then*, *after*, *because*), intonational patterns (e.g., flat intonation, atypical pitch contours), and speech style markers (e.g., contextually appropriate use of dialect or formal registers). These components are closely tied to higher-order linguistic processing and cognitive planning. Notably, older adults may exhibit reduced scores on such measures, potentially reflecting age-related changes in lexical retrieval, syntactic complexity, and working memory demands required for constructing coherent and structured discourse^(33,35)^.

Non-propositional aspects, on the other hand, relate to discourse assessments, including revision behaviors (such as false starts, reformulations) and repair methods (such as repetitions and self-corrections). Even though these characteristics are frequently compensatory, they nonetheless necessitate precise control over the production and monitoring of discourse, especially across various discourse types (e.g., narrative, conversational, procedural). A more sophisticated comprehension of the linguistic and cognitive constraints imposed by different discourse types is made possible by the incorporation of both propositional and non-propositional criteria. The inclusion of both propositional and non-propositional parameters in the assessment allows for a better understanding of the linguistic and cognitive demands imposed by different discourse types. Such comprehensive linguistic metrics should be used in future studies to more accurately describe the discourse-level changes associated with healthy ageing.

Within each age cohort, distinct performance was observed across the discourse types. The study’s findings revealed that performance on the picture description task was better than in conversation and narration among both young and middle-aged adults. However, among older adults, performance in conversation was superior to the other two discourse types (narrative discourse and picture description). This finding aligns with the reports of Babaei et al.^(16)^.

Two reasons could explain this finding. Firstly, picture description involves the visual processing of the target lexical items, followed by retrieving the semantic and phonological forms to produce the target word. This process could place constraints on and limit opportunities for variations in the number of lexical items that can be used in connected speech, which is a factor that can be considered under the aspect of individual differences. Secondly, there are no contextual cues available to the individual, unlike those present in other discourse types^(36)^. In contrast to these findings, a study^(37)^ posited that the picture description task is more cognitively demanding than the conversation and narration tasks. The augmented cognitive load in picture description as compared to narration and conversation tasks may be attributed to the need to retrieve the exact items depicted in the pictures and to narrate the events with precision. Despite the robust findings, these findings should be interpreted with caution, owing to the small sample size.

Conversation, specifically, provides pre-established mental models and analogical representations that facilitate the analysis and interpretation of received information^(38,39)^, which may help older individuals perform relatively better than in other tasks. Additionally, the authors speculate that conversation is an activity that is relatively and extensively used in day-to-day life. Over time, older individuals have regularly engaged in this activity and gained familiarity, which may have led to their better performance in conversation compared to other tasks. Although different age cohorts are greatly influenced by distinct discourse types, it is still challenging to ascertain if these discourse patterns are exclusively the result of ageing due to their intrinsic complexity and character. Hence, future research should build on the current study’s findings to establish a more robust evidence base in this area.

Bilingualism may be advantageous for performing better on these tasks^(40,41)^, but this conclusion could not be explicitly stated in the current study due to the lack of a monolingual cohort. Future research should compare the performances of monolinguals and bilinguals to confirm this finding. Additionally, factors such as language dominance, task demands, and age may influence discourse performance, with picture description being the most affected as it relies heavily on lexical access^(42)^, which declines with age and is susceptible to bilingual interference.

The present study investigated discourse production in Konkani–Kannada bilinguals. Given the widespread occurrence of multilingualism across the globe, analogous discourse patterns, including better narrative and conversational abilities with advancing age and greater susceptibility in picture description, may indicate shared cognitive mechanisms among bilingual speakers. As discussed earlier, these phenomena correspond with the advantages of bilingualism in executive control and the age-related challenges of lexical access. It is important to note that language-specific factors such as dominance, proficiency, and age of acquisition can influence performance; however, the cognitive demands associated with managing multiple languages are comparable across various contexts. Therefore, the current findings may be parallel with other multilingual populations, although further validation across diverse language pairs is essential to ascertain their generalizability.

Discourse production differed between genders in the current study, with females demonstrating higher scores in both conversation and picture description tasks. This finding is consistent with the results of Wainwright’s study^(43)^. Other studies have also reported gender-related differences in narrative production abilities; for example, Schulkind et al.^(44)^ observed qualitative differences in autobiographical narratives. Overall, gender differences in discourse production are well-documented in the literature. However, the present study did not examine qualitative differences and their performance across discourse types, which could have provided a deeper understanding of their performance. This represents one of the limitations of the study.

From this study, multiple key points can be drawn, such as the utility of the type of discourse across the age group, i.e., young, middle, and older adults. However, the picture stimulus would provide a live cue for the referent that has to be retrieved from the lexicon^(6,45)^ and involves a different processing style compared to narration and conversation, which places more demands on the processing system, as it more taps on the attention, memory and executive functions as in for retrieval, formulation and production of continuous speech (in narration and conversation). Hence, there could be better performance on picture description over the other two tasks in young and middle-aged adults. However, older adults could not utilize the available visual aids for the formulation of discourse compared to other groups. Gaining an expanded understanding of how language production skills evolve throughout healthy ageing is essential to identifying variations that could be linked to pathological ageing^(46)^. Hence, it is important to consider these aspects while selecting the type of task and assessing the discourse abilities to dissociate the performance across the age groups and pathological conditions.

Though this is the first study to explore discourse performance in Konkani-Kannada bilinguals, there are some limitations that future research could address. Disruptions in fluency and lexical measures can be included as additional metrics to assess discourse performance. Instead of using a single picture, sequential pictures could be employed, as they involve logical and temporal sequences that allow for better establishment of cohesion. This approach might help older individuals perform better in this type. However, Babaei et al.’s study^(16)^ found the opposite outcome, highlighting the importance of replicating study results. Furthermore, it is crucial to extend comparisons to clinical groups and between different clinical groups. Additionally, it would be intriguing to identify which factors are most relevant to this form of evaluation and are strongly associated with deficits in cognitive capacities, such as executive function, memory, and attention.

Although the study is well-designed and controls for the extrinsic variable affecting ageing, it has certain limitations in the way discourse is analyzed. Quantitative parameters that involve microstructures, such as mean length of utterances, type token ratio, number of pauses, fluency, etc, can be considered. The authors in the study mainly assessed discourse qualitatively, but incorporating quantitative measures could have given a deeper understanding of the discourse sample in general. The categorization of groups was mainly done based on age and cognitive scores, but further studies can benefit from creating cohort groups based on education, occupation, and socio-economic status.

## CONCLUSION

The results of this study demonstrate that discourse is influenced by both ageing and the type of discourse. The older adults group faced greater challenges with narrative discourse, suggesting that the cognitive and linguistic demands of constructing coherent narratives may be more sensitive to age-related changes. These findings may assist speech-language pathologists in considering various factors during assessment and management programs for older individuals. Tailoring assessment protocols to include a range of discourse tasks can provide a more comprehensive picture of an individual’s communicative strengths and challenges. Furthermore, intervention programs might benefit from targeted support in narrative skills, which appear particularly vulnerable to age-related decline. Overall, this study underscores the need for age- and discourse type-sensitive approaches in both assessment and therapy planning for the ageing population.
